# Phylogeography of Phytophagous Weevils and Plant Species in Broadleaved Evergreen Forests: A Congruent Genetic Gap between Western and Eastern Parts of Japan

**DOI:** 10.3390/insects2020128

**Published:** 2011-04-21

**Authors:** Kyoko Aoki, Makoto Kato, Noriaki Murakami

**Affiliations:** 1 Graduate School of Human and Environmental Studies, Kyoto University, Kyoto, Kyoto 606-8501, Japan; E-Mail: kato@zoo.zool.kyoto-u.ac.jp; 2 Makino Herbarium, Tokyo Metropolitan University, Hachioji, Tokyo 192-0397, Japan; E-Mail: nmurak@tmu.ac.jp

**Keywords:** comparative phylogeography, glacial refugia, biogeography, population expansion, host specificity, parasite, *Castanopsis*, *Curculio*, *Rhynchaenus*, Japan

## Abstract

The Quaternary climate cycles played an important role in shaping the distribution of biodiversity among current populations, even in warm-temperate zones, where land was not covered by ice sheets. We focused on the *Castanopsis*-type broadleaved evergreen forest community in Japan, which characterizes the biodiversity and endemism of the warm-temperate zone. A comparison of the phylogeographic patterns of three types of phytophagous weevils associated with *Castanopsis* (a host-specific seed predator, a generalist seed predator, and a host-specific leaf miner) and several other plant species inhabiting the forests revealed largely congruent patterns of genetic differentiation between western and eastern parts of the main islands of Japan. A genetic gap was detected in the Kii Peninsula to Chugoku-Shikoku region, around the Seto Inland Sea. The patterns of western-eastern differentiation suggest past fragmentation of broadleaved evergreen forests into at least two separate refugia consisting of the southern parts of Kyushu to Shikoku and of Kii to Boso Peninsula. Moreover, the congruent phylogeographic patterns observed in *Castanopsis* and the phytophagous insect species imply that the plant-herbivore relationship has been largely maintained since the last glacial periods. These results reinforce the robustness of the deduced glacial and postglacial histories of Castanopsis-associated organisms.

## 1. Introduction

Phylogeographic patterns of extant genetic variation in organisms have proven to be highly informative in recovering their postglacial demographic histories [1,2]. Another powerful method is comparing the intraspecific phylogeographic patterns among several taxa over the same area and searching for congruent geographic patterns of genetic variation, which indicate the influence of common historical factors [3–16]. Comparing the intraspecific phylogeographic patterns among different species distributed within a single vegetation zone should be more informative because a group of species living together in the present environment are likely to have responded similarly to past geological and climatic events [3,17]. In the present study, we focused on the broadleaved evergreen forest community in Japan that characterizes the biodiversity and endemism of the warm temperate zone.

**Figure 1:**
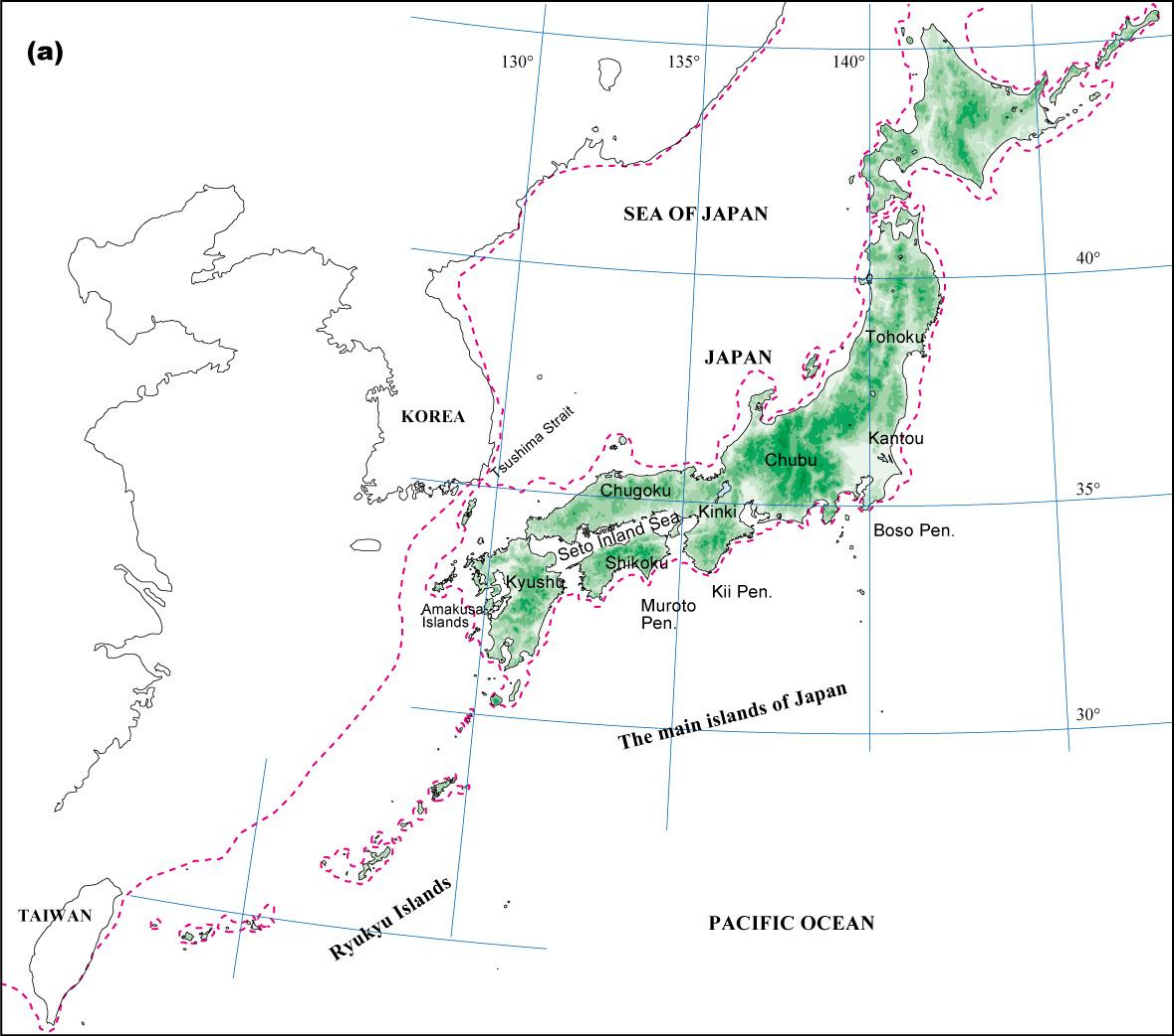

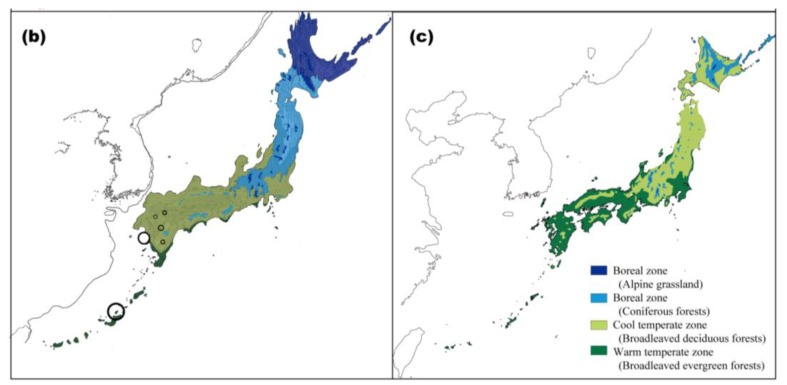
The location and vegetation of Japan. (a) The location of the Japanese Archipelago. The dotted line indicates the coastline of the Last Glacial Maximum about 20,000 years ago. (b) Vegetation during LGM based on Kamei and Research Group for the biogeography from Würm Glacial [29]. Circles indicate the existence of pollen records of broadleaved evergreen trees at the LGM [22,30]. (c) Potential natural vegetation at present [31].

Climatic changes during glacial periods have had a major influence on the recent evolutionary history of living organisms, even in the warm-temperate zone, where land was not covered by ice sheets [18,19]. The geological and geographical features of the Japanese Archipelago consist of several mountain ranges running parallel to a northeast-southwest-oriented axis. As the coastal belt is close to these mountain ranges, the climate varies even within a narrow region. Consequently, various types of vegetation occur in the archipelago (Figure 1a). Moreover, several landbridges between Japan and its surrounding areas, which formed or disappeared in response to glacial-interglacial climatic changes, have played important roles in determining the current distribution of the biological diversity in Japan [20]. Current warm-temperate and subtropical zones in the Japanese Archipelago are covered with forests mainly dominated by three types of broadleaved evergreen trees: *Castanopsis, Quercus,* and *Machilus* [21].

Palynological evidence indicates that broadleaved evergreen forests in Japan were subjected to cold periods at least four times during the Quaternary [18,19]. Throughout the glacial periods, climatic cooling caused southward and toward lower altitudes shifts in the geographic distribution of these forests. The pollen record indicates that refugial populations of broadleaved evergreen forests were restricted to southern areas, mainly at the southern end of Kyushu, and migrated northward from the refugia after the Last Glacial Maximum (LGM) (see Figure 1b,c and [19,22,23]).

In this study, we used molecular markers to compare the intraspecific phylogeographic patterns of several plant and insect species present in a Japanese *Castanopsis*-type broadleaved evergreen forest community. Plant chloroplast DNA (cpDNA) is effectively haploid with a small effective population size and is usually maternally inherited and thus often used in molecular phylogeographic studies. However, the molecular evolutionary rate of cpDNA has been reported to be relatively slow at the nucleotide sequence level [24–26]. We first surveyed the intraspecific variation among many component species of broadleaved evergreen forests using nucleotide sequence analyses of cpDNA. We aimed to identify the species that had a relatively large incidence of intraspecific cpDNA variation [27,28]. Eight species growing in *Castanopsis*-dominant forests were selected for the phylogeographic study. Furthermore, we used microsatellite markers (expressed sequence tags-simple sequence repeat: EST-SSR) from *Castanopsis* owing to its extremely low levels of intraspecific variation in cpDNA.

*Castanopsis* trees are associated with diverse herbivorous insects. We investigated phylogeographic patterns based on the current geographic distribution of the informative mitochondrial DNA (mtDNA) diversity among three types of the weevils associated with *Castanopsis*: the host-specific seed-boring weevil *Curculio hilgendorfi*, the generalist seed-boring weevil *Curculio sikkimensis*, and the host-specific leaf-mining weevil *Rhynchaenus dorsoplanatus*. The phytophagous beetle species has an advantage of low dispersability, e.g., only flying to oviposition substrates (*i.e.*, seeds and new leaves). Moreover, these almost flightless and nonmigratory insects likely responded to environmental changes associated with the glacial cycles in a manner similar to their host plants. Thus, we compared the genetic structures of these weevils with those of *Castanopsis*. If the plant-insect association (*i.e.*, the host range of the weevil) remained stable throughout the Quaternary, the presence of the phytophagous insects may be an indicator of the coverage of the associated forests. Thus, incorporating phylogeographic information of phytophagous insect species into that of the host plant species could elucidate more precisely the phylogeographic patterns of the inhabited forests.

In this paper, we reviewed and compared the phylogeographic patterns of plant and insect species inhabiting the *Castanopsis*-type broadleaved evergreen forests in Japan. This study addressed whether geographic patterns of genetic variation are congruent between the plant species growing in *Castanopsis*-type forests and whether phylogeographic patterns are similar among the host plants *Castanopsis* and the three types of the weevil species that share the same host. The phylogeographic patterns of these weevil species will enable us to reconstruct the glacial and postglacial history of the associated *Castanopsis* forests in Japan.

## 2. Experimental Section

### 2.1. Plant Species

We first surveyed the intraspecific variation among 61 component species of broadleaved evergreen forests in Japan and the surrounding areas using the nucleotide sequence analyses of cpDNA. Among these species, intraspecific cpDNA variation was detected in 17 plant species. We selected eight species belonging to distantly related plant families that inhabit *Castanopsis*-dominant forests.

Seven species out of the eight plant species, such as *Elaeocarpus sylvestris* var. *ellipticus*, *Prunus zippeliana*, *Myrsine seguinii*, *Daphne kiusiana*, *Alpinia japonica*, *Arachniodes sporadosora*, and *Arachniodes aristata*, have a similar geographic distribution pattern (along the Pacific coast up to the Boso Peninsula, known as the northern limit of the distribution), but represent a diverse array of life histories (*i.e.*, ferns, perennials, shrubs, and trees) and dispersal modes (*i.e*., wind and birds) (Table 1). Our sampling sites covered practically the entire range of distribution of these plant species throughout Japan. The current geographic distribution of *Photinia glabra* is different from those of the above-mentioned species, occurring primarily in Kinki and the eastern Shikoku and Chugoku regions. Disjunct populations are known in the Amakusa Islands of southwestern Kyushu. Sequencing of noncoding regions of cpDNA and data analyses are described in previous research cited in Table 1.

We investigated the phylogeographic patterns of *Castanopsis* by analyzing EST-SSR markers [38–41]. The plant genus *Castanopsis* in Japan consists of two species, *Ca. cuspidata* and *Ca. sieboldii*, and the latter is divided into two varieties, var. *sieboldii* and var. *lutchuensis* [42,43]. *Castanopsis cuspidata* and *Ca. sieboldii* var. *sieboldii* are distributed among the main islands of Japan and *Ca. sieboldii* var. *lutchuensis* is only found within the Ryukyu Islands. *Castanopsis cuspidata* and *Ca. sieboldii* var. *sieboldii* are sometimes distributed sympatrically in the main islands and can be morphologically distinguished by differences in their seed size, shape, and the structure of their leaf epidermis [42,44]. *Castanopsis sieboldii* var. *sieboldii* has large oblong seeds and exhibits two epidermis cell layers, whereas *Ca. cuspidata* has small globular seeds and one epidermis cell layer. Intermediate morphological types have been frequently reported, especially at sites where the two species coexist [45,46]. EST-SSR analyses based on Ueno *et al.* [38] were performed and an unweighted pair group method with arithmetic mean (UPGMA) dendrogram was constructed based on Nei's genetic distance [47].

### 2.2. Insect Species

We investigated the genetic variation in mtDNA of three types of weevils (Coleoptera: Curculionidae): *C. hilgendorfi*, *C. sikkimensis*, and *R. dorsoplanatus*, which are associated with *Castanopsis* trees. *Curculio hilgendorfi* is an obligate seed predator whose larvae feed specifically on the seeds of *Ca. sieboldii* in Japan, whereas *C. sikkimensis* is a generalist seed predator associated with deciduous and evergreen Fagaceae trees [*i.e.*, *Quercus* (deciduous and evergreen), *Lithocarpus* (evergreen), *Castanopsis* (evergreen), and *Castanea* (deciduous)]. *Rhynchaenus dorsoplanatus* is a host-specific leaf miner of *Castanopsis* (*i.e.*, *Ca. sieboldii* var. *sieboldii* and *Ca. cuspidata*).

The female *Curculio* drills into *Castanopsis* seeds with extremely long rostra and oviposits in the pore during autumn. The larvae feed on the seeds prior to being left to overwinter in the soil, pupating before they emerge in spring or summer. New *Castanopsis* leaves are bored by female *Rhynchaenus* with their short rostra and oviposit in the perforation during spring. Palisade and spongy parenchyma of the leaves are consumed by the larvae. In late spring, adult weevils emerge from the leaves. Our sampling sites covered practically the entire range of distribution of these weevil species throughout Japan. Sequencing of mtDNA and data analyses are described in previous research cited in Table 1.

## 3. Results and Discussion

### 3.1. Common Phylogeographic Patterns among the Broadleaved Evergreen Plant Species

#### Genetic structure

**Figure 2:**
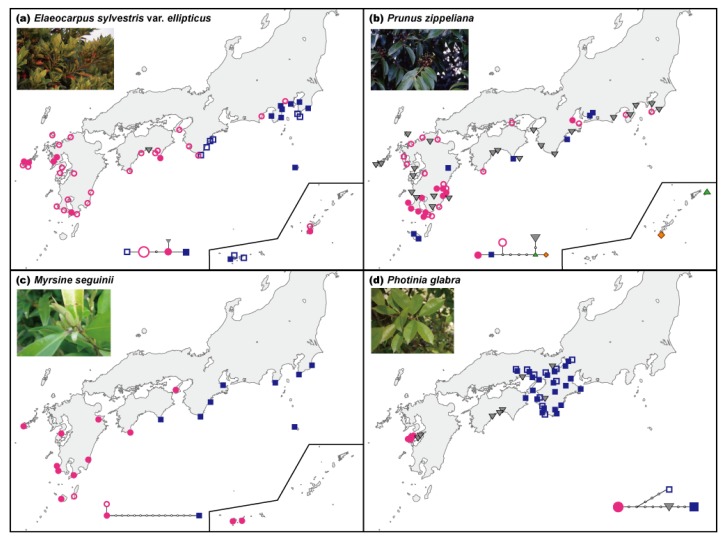

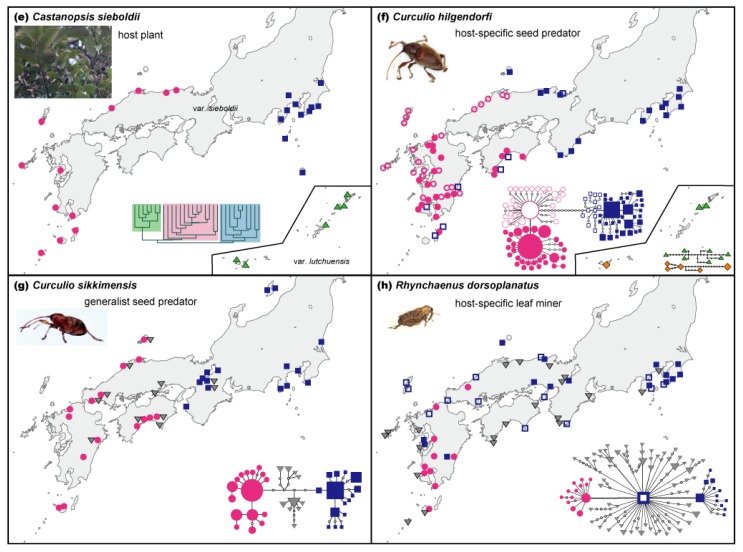
The western-eastern differentiated pattern observed in the main islands of Japan. Many plants and insects inhabiting *Castanopsis*-type broadleaved evergreen forests share this pattern with a major phylogeographic break typically occurring at various points in the Kii Peninsula to Chugoku-Shikoku region around the Seto Inland Sea (modified from references shown in Table 1). (a–d) Geographic distribution of cpDNA haplotypes found among four plant species of broadleaved evergreen trees. (e) Geographic distribution of the UPGMA clusters found among EST-SSR markers of *Ca. sieboldii*, the dominant tree. (f–h) Geographic distribution of mtDNA clades found among three weevil species associated with *Castanopsis*.

The geographic distribution patterns of cpDNA haplotypes from several broadleaved evergreen species showed no clear geographical structuring (Table 1). The molecular evolutionary rate of cpDNA has been reported to be relatively slow at the nucleotide sequence level [24–26]. In fact, the molecular evolutionary rate of noncoding cpDNA regions (*trnT-trnL*, *trnL-trnF*, *atpB-rbcL*, *rps16*, *trnG*, *psbC-trnS*, *trnG-trnfM*, *trnW-trnP*, *petB*, *petD-rpoA*, *rpl16*) that are often used for phylogeographic analyses is also slow, *i.e.*, 1.2 × 10^−10^ (including indels) and 7.7 × 10^−11^ (nucleotide substitutions only) substitutions per site per year [32], or one nucleotide substitution every two million years per site on average. In general, relatively low levels of cpDNA variation are usually found within species. Moreover, extremely low levels of intraspecific variation in cpDNA were reported in Japanese broadleaved evergreen species [27,28] than in plants growing in other vegetation zones, *i.e.*, broadleaved deciduous species [48] and alpine species [49]. This result suggests that the effects of climate change are particularly severe for members of the broadleaved evergreen forests community in the warm-temperate zone. Nevertheless, the geographic distribution patterns of cpDNA haplotypes in several plant species (Table 1, Figure 2a,c,d) and the clusters found among the EST-SSR variation of *Ca. sieboldii* (Figure 2e) clearly differentiated populations between the western and eastern parts of the main islands of Japan. Moreover, *P. zippeliana* (Figure 2b) and *Ca. sieboldii* (Figure 2e) showed genetic uniqueness in the Ryukyu Islands. This apparent geographical structuring was observed in the tree species, especially in the dominant species, rather than in herbs or ferns.

#### Genetic uniqueness and genetic diversity

We compared the intraspecific phylogeographic patterns among six plant species (*P. zippeliana*, *A. japonica*, *D. kiusiana*, *E. sylvestris* var. *ellipticus*, *A. sporadosora*, and *A. aristata*; see Table 1 for materials) inhabiting the broadleaved evergreen forests with respect to cpDNA haplotype uniqueness and haplotype diversity [3]. Many rare haplotypes and the greatest numbers of haplotypes were observed in the Ryukyu Islands and Kyushu, a finding that agreed with fossilized pollen data demonstrating the past existence of refugia in the Ryukyu Islands [30] and southern Kyushu (see Figure 1b and [19,22]).

Note that several rare types were observed on the Muroto Peninsula and that the number of common haplotypes was also high on the Kii Peninsula. Because the Muroto Peninsula was contiguous with the Kii Peninsula during the LGM (see Figure 1b and [19,29]), we also hypothesized that additional important refugia existed from the Muroto to Kii peninsulas during the glacial periods, where pollen records of broadleaved evergreen species are scarce. Moreover, the phylogeographic pattern of cpDNA in *P. glabra* with a current disjunctive geographic distribution also supports the existence of the two refugia of southern Kyushu and around the Kii Peninsula [35].

With regard to the relatively slow rate in molecular evolution of cpDNA discussed above, most of the cpDNA haplotypes found in one plant species probably originated long before the Quaternary period. The geographic distribution of most Japanese broadleaved evergreen species was probably restricted to the two refugia of the western and eastern parts during repeated glacial periods in the Quaternary period. After climate warming, since the LGM about 20,000 years ago, these surviving populations may have expanded very slowly and have not come into contact with some species as of yet.

### 3.2. Common Phylogeographic Patterns among Host Plant *Castanopsis* and the Weevil Species

#### Genetic structure

A comparison of the genetic structures between the host plant and insects revealed that the patterns of genetic differentiation are largely congruent in the western and eastern parts of the main islands. Moreover, congruent phylogeographic patterns were observed among the three weevils (see Figure 1f–h and [33,34,36]), with a gap between the western and eastern clades on the main islands with respect to the mtDNA sequences. The observed gap between the western and eastern clades of these weevils suggests that the western and eastern insect populations have been isolated for a long time, probably through several glacial and interglacial periods; the genetic groups have not been in contact for several thousands of years since the last glacial period, and long-distance migration of the weevil has been rare, resulting in negligible interpopulation genetic exchange events. This result suggests past fragmentation of these weevils into at least two separate regions. Among these weevils, only *C. hilgendorfi* is distributed in the Ryukyu Islands. The phylogenetic tree and haplotype network of *C. hilgendorfi* showed that Ryukyu populations have clearly differentiated genetically from those of the main islands of Japan (see Figure 2f and [33]), which is also demonstrated by the patterns of the host plant *Ca. sieboldii* (Figure 2e).

The broad-scale congruent phylogeographic pattern of western and eastern separation in the main islands observed among the weevils suggests that they experienced similar historical and environmental changes. Some differences in the patterns were also observed among these species. A larger gap of the western and eastern clades and a higher genetic differentiation between these areas were observed in *C. hilgendorfi*, whereas low genetic differentiation between the clades and the existence of several clades in the intermediate areas of Chugoku-Shikoku were detected in *C. sikkimensis* and *R dorsoplanatus*. Moreover, genetic differentiation was noted in the western clade of *C. hilgendorfi* between the coasts of the Pacific and the Sea of Japan, which suggested the existence of additional important refugia in the northwest part of Kyushu in addition to the southern end of Kyushu.

These distinctions in the extent of the genetic differentiation may have been affected by differences in host specificity and plant parts infested by weevil species, jointly with flight ability and differences in life history. Among these weevil species, *C. hilgendorfi* has the strongest host specificity (*i.e.*, one-to-one association). The lack of distribution of the host plant *Ca. sieboldii* around the Seto Inland Sea may have prompted genetic differentiation between *C. hilgendorfi* on the Pacific coast and those on the coast of the Sea of Japan. In contrast, *C. sikkimensis* is a generalist seed predator of Fagaceae plants and could migrate across the Seto Inland Sea using several of these plant species in the Chugoku-Shikoku regions. This polyphagous habit allowed *C. sikkimensis* to cross the Seto Inland Sea and may have generated intermediate clades in the Chugoku-Shikoku region.

The weight of the leaf miner *R. dorsoplanatus* is about one-tenth that of *C. hilgendorfi*, and consequently, the former might have been transported by scarce strong winds such as a typhoon. Dispersal of larvae through the drifting of seeds or leaves containing the larvae is not likely to occur in either species. If gene flow in *R. dorsoplanatus* occasionally occurred by long-distance dispersal among isolated populations in the glacial refugia, the genetic differentiation between isolated refugia was not promoted. Weak genetic differentiation in *R. dorsoplanatus* may be explained by dispersion due to its small body size.

The seed-boring *Curculio* overwinters underground during its larval stage and has greater cold tolerance than *R. dorsoplanatus*, which overwinters at the stage of imago on the ground. Cold tolerance may have enabled *Curculio* to survive in isolated populations for a long time, probably through several glacial and interglacial periods, and consequently, it shaped the large genetic gap between the western and eastern clades. In addition, seeds are a more unpredictable resource than new leaves because the number of seeds available for predation greatly fluctuates annually and supra-annually [50,51]. For example, during a poor crop year, the population size of *Curculio* must have been gravely reduced, resulting in a population bottleneck. These ecological characteristics of the seed-parasitic weevil species may have caused stronger genetic structuring than in the leaf-mining weevil species.

The results suggested that broad-scale concordance of the phylogeographic patterns may have been formed by the common historical and environmental changes experienced by the weevils, whereas the local-scale discordance may have occurred by differences in the host specificity and ecological characteristics of the weevils. A more detailed explanation of what formed the local variations in these patterns can be estimated by comparing them to various phytophagous insects with diverse geographic distributions and life histories associated with *Castanopsis*.

#### Demography

We compared the demographic history of the two host-specific weevil associated with *Castanopsis*, *C. hilgendorfi* and *R. dorsoplanatus*. The expansion events of both weevil species in the main islands were dated approximately to 40,000–100,000 and 30,000–120,000 years ago, inferred from the mismatch distributions and coalescent-based Bayesian skyline plots, respectively [36]. Molecular dating based on a single locus should obviously be interpreted with caution. Nevertheless, these data suggested that the beginning time of these expansion events is consistent with the last glacial periods (120,000–12,000 years ago, [52]), when it was cooler than at present. The congruent time of the population expansion events of the two host-specific predators suggest that the associated *Castanopsis* forests experienced a reduction in population size during the last cooling. In *C. hilgendorfi*, large sequence diversity and multimodal mismatch distribution of sequence differences in the Ryukyu Islands populations were observed [33]. This suggests that historically, these populations have been large enough for ancestral polymorphism to be retained for a long time.

### 3.3. Glacial and Postglacial History of Broadleaved Evergreen Forests

#### Genetic uniqueness and higher genetic diversity observed in the Ryukyu Islands

The phylogeographic patterns of several plant species and *C. hilgendorfi* showed that populations found in the Ryukyu Islands have genetically differentiated from those of the main islands of Japan. Many rare haplotypes and the greatest numbers of haplotypes among plant species were observed in the Ryukyu Islands, although unique haplotypes were not detected in some plant species. Such genetic uniqueness and high genetic diversity were also found in several plant [10,53] and animal [54,55] species cohabiting in warm-temperate and subtropical zones in Japan. The Ryukyu Islands have been isolated from Kyushu since 1,500,000 years ago [56]. The genetic differentiation observed in these islands is suggested to be promoted by the long-term restriction of gene flow from the main islands. Indeed, the pollen records indicate the existence of broadleaved evergreen trees at the LGM in the Ryukyu Islands (see Figure 1b and [30]).

The large sequence diversity and demographic equilibrium in *C. hilgendorfi* suggest that the Ryukyu Islands populations have not experienced a rapid range expansion or serial population bottleneck during recent glacial periods. On these islands, insects associated with broadleaved evergreen forest trees could probably have survived in large populations throughout the entire duration of the glacial periods based on their location far to the south, which had a much warmer climate than that of the main islands.

#### Genetic differentiation between the western and eastern parts of the main islands

The genetic differentiation between the western and eastern populations observed among many plant and insect species in *Castanopsis*-type broadleaved evergreen forests suggests that the forests have been isolated from the western and eastern populations for an extended time, probably through several glacial and interglacial periods. Such a boundary is also found in several other plant species growing in warm-temperate zones in Japan (Table 2).

In Europe, the Alps and Pyrenees may have blocked the dispersal of many animal and tree species from Italian and Iberian refugia to the northern part of Europe (reviewed in [8,15]). However, in the case of the geographical boundary in the Chugoku-Shikoku region, neither a north-south mountain range nor a temperature gap has been observed. Other geographical and climatic features of the Seto Inland Sea, such as a currently drier climate and limited onshore wind, and the historical existence of a grassland landscape during dry cool climates in the glacial ages may have served as barriers against recolonization of various species from their refugia. The appearance of the inland sea between the Kii Peninsula and Shikoku region after the glacial periods has also served as barriers against recolonization from their refugia.

The following scenarios were deduced from these data: Japanese broadleaved evergreen forests in the mainland survived in the western, probably at the south end and northwestern part of Kyushu (from the data of *C. hilgendorfi* described in Section 3.2), and the eastern (probably around the Kii Peninsula from the data of several plants described in Section 3.1) parts during the last glacial period; and after climate warming, these surviving small forests may have expanded very rapidly along the coast from west to east.

In this study, *C. sikkimensis* inhabiting both broadleaved deciduous and evergreen forests and *C. hilgendorfi* associated only in broadleaved evergreen forests with several other plant species, shared a common phylogeographic pattern with both exhibiting a genetic gap between the eastern and western parts of Japan (Figure 2f,g). The geographic distributions of the genetic diversity of plant species and their parasitic insects suggest that both were restricted to separate western and eastern refugia on the main islands of Japan during repeated glacial periods in the Quaternary. Moreover, the geographical boundary in the Kii Peninsula (Kinki) to the Chugoku-Shikoku region was also observed in several other plant and animal species from warm- and cool-temperate zones of Japan (Table 2).

At the LGM, the global sea level is estimated to have dropped to about 140 m lower than the present level [80]. The narrowing of the Tsushima Strait at that time caused a reduction in the Tsushima Warm Current into the Sea of Japan, resulting in further cooling and the aridification of the Japanese Archipelago. Given this cool and arid climate, the areas covered by coniferous forests and cool-mixed forests expanded southward [81]. This suggests that both deciduous and evergreen forests were restricted to adjacent refugia at that time. The coincident patterns of *C. sikkimensis* and *C. hilgendorfi* strongly support the hypothesis that both deciduous and evergreen forest types survived together or adjacent to one another in small refugia during the glacial ages, although these forests are presently separately distributed in cool- and warm-temperate zones, respectively. The geographical boundary observed by many phylogeographic studies on the species in cool-temperate zones appears farther east of that of the species in warm-temperate zones. For example, the Chubu boundary was observed in many species from cool-temperate zones, whereas the Chugoku-Shikoku boundary was observed in many species from warm-temperate zones (Tables 1,2). A more detailed history can be estimated by comparing the phylogeographic patterns of various organisms with diverse life histories in these types of forests.

In some insect species from Japanese cool- and warm-temperate forests, the pattern of west-east differentiation has not been observed. For example, with regard to species interaction, the offensive trait of the weevil and the defensive trait of the plant are involved in a geographically structured arms race, suggesting the overwhelming strength of coevolutionary selection against the effect of historical events that may have limited local adaptation [82]. The divergence of flightless ground beetles (*Ohomopterus*) is suggested to have occurred with the varying fragmentation of favorable habitats by geographical barriers, evolution of body size, and genital morphology in local populations, and secondary contact and interactions between diverging populations [83]. The larvae of the beetle are specialized predators of megascolecid earthworms, and *Ohomopterus* populations depend on the presence of these earthworms. This non-phytophagous species appears to have its own distinctive evolutionary history, and the effects of historical climatic change at the vegetation scale were not significant on their present geographical distribution. Our data suggest that the continual historical change of the environment on Japanese broadleaved evergreen forests has contributed to shaping the recent genetic structure of phytophagous insects.

### 3.4. Phytophagous Insects Reveal Recent Evolutionary Histories of Their Host Plants

Whiteman and Parker [84] and Nieberding and Olivieri [85] summarized the theoretical framework for using parasites as proxies for their host evolutionary history. Parasites are most useful to complement host genealogy when host genetic data retain excessive ancestral polymorphisms or lack of population structure (e.g., species with low genetic variability owing to recent strong bottlenecks, endangered species, or invasive species [85]). Moreover, parasites with short generation times and high mutation rates, such as viruses, are most informative for deciphering recent host demographic events, because they can accumulate sufficient mutations in the short time periods [86].

Most plant species are associated with diverse host-specific phytophagous insects. In the case of a pair of the host plants and their host-specific phytophagous insects, the synonymous rate of sequence change has been estimated to be considerably faster in animal mtDNA than in plant cpDNA, mtDNA, or even nuclear DNA [26,87–89]. With their shorter generation times and more quickly evolving genomes, phytophagous insects can be extremely useful tools for clarifying previously undetected historical events of their host plant. To apply genetic information of insects to their host plants, the phytophagous insects should be highly likely to have shared a common history with its host and to display a more resolved genetic pattern.

At the phylogenetic scale, several studies suggest that co-divergence of host plants and obligate pollination mutualistic insects have occurred [90–92]. Several tests for analyzing the level of co-speciation between phylogenies indicate that a greater degree of correlation generally exists between host trees and their seed-parasitic pollinator phylogenies than expected from a random association [91].

At the intraspecific level, very few studies have compared the phylogeographic patterns or genealogies between plant species and their specialized herbivorous insect species. An alpine plant species and its specialized herbivorous insect, the butterfly, from the Rocky Mountains suggest that they respond similarly, but independently to climate cycles [93]. Incongruent patterns of genetic variation and independent biogeographic histories in the specialized plant-insect association suggest that by promoting habitat expansion and mixing among alpine populations, glacial periods repeatedly reset the distributions of genetic variation in each species and inhibited continual co-divergence among pairs of interacting species. The similar host and parasite genealogical tree has been scarcely observed in the specialized plant-insect association. In contrast, in the case of host-parasite relationships, partial spatial and temporal congruences in the differentiation of both field mice and their specific parasites (nematodes) were revealed [94]. The lack of or low levels of intraspecific variation in plants and high dispersability of insect species by flying may prevent the detailed phylogeographic congruence between plants and insects.

Parasite features shown to determine the strong likelihood of a parasite sharing a common history with its host are the level of intimacy of the interaction between the two organisms: host specificity, the lack of intermediate host and a free-living phase, or mutualistic symbionts [84–86]. The existence of a free-flying phase in the imaginal stage might decrease the dependence of the insects on its host plants, even though insect species have high host-specificity. Subsequently, the assumption of intimate host-parasite relationships seems more difficult to demonstrate with phytophagous insects than with parasites. We should then select host-specific phytophagous insect species for the biogeographic study of host plants, such as almost flightless and nonmigratory or with a shorter imaginal stage.

Our study suggests that the geographic patterns of the genetic differentiation between western and eastern parts of the main islands are largely congruent between the host plant and its phytophagous insects. We could not directly compare the genealogical trees of the insects to its host plant because we did not perform the population analysis of the insect mtDNA variations. If the genealogical trees of the host plant and phytophyagous insects are identical in future studies, their comparisons suggest parallel histories of *Castanopsis* and its phytophagous insect populations during and after the last glacial periods. The investigation of recent phylogeographic processes directly from plant species that infer the historical dynamics, locate glacial refugia in the Quaternary, and reveal detailed postglacial colonizing routes has been somewhat limited due to the relatively slow rate in molecular evolution of cpDNA in plants and extremely low levels of intraspecific variation in cpDNA of Japanese broadleaved evergreen species. The use of DNA markers from insects with a faster evolutionary rate can make possible the reconstruction of previously undetected evolutionary and demographic histories of their host plants and associated forests. Indeed, analyses of *C. hilgendorfi* detected another refugial area (*i.e.*, northwestern part of Kyushu) that was not previously recognized by analyzing intraspecific cpDNA variation of several plant species inhabiting the *Castanopsis*-type broadleaved evergreen forests (described in Section 3.2 and [33]). The congruent phylogeographic patterns that were observed in the weevil species reinforce the robustness of the deduced glacial and postglacial histories of *Castanopsis*-associated organisms.

## 4. Conclusions

Our data suggest that geological and historical environments have contributed to shaping the recent genetic structure of many plant and insect species inhabiting *Castanopsis*-type broadleaved evergreen forests of Japan. Comparing phylogeographic patterns of three types of phytophagous weevils (*i.e.*, a host-specific seed predator, a generalist seed predator, and a host-specific leaf miner), and of several plant species, enabled us to specify the historical processes of these forests more precisely. We demonstrated that these insects and their host plant were restricted to separate western and eastern refugia on the main islands of Japan during repeated glacial periods in the Quaternary. The congruent phylogeographic patterns that were observed in the weevil species associated with the same host plant reinforce the robustness of the deduced glacial and postglacial histories of *Castanopsis*-associated organisms. Our analyses provide a foundation for exploring the evolutionary history of Japanese broadleaved evergreen forests, which characterize the biodiversity and endemism of the cool- and warm-temperate zones severely affected by climate change.

## Figures and Tables

**Table 1:** Population studies based on genetic markers from plant and insect species inhabiting Japanese *Castanopsis*-type broadleaved evergreen forests [3,27,32–37].

**Species**	**Family**	**Life Form**	**Dispersal Form**	**No. of Populations**	**No. of Individuals**	**Genome Markers**	**Total Length (bp)**	**Gene Diversity (*h*)**	**Nucleotide Diversity (p)**	**Phylogeographic Pattern (Geographic Gap)**	**References**
**Plants**											
*Elaeocarpus sylvestris* var. *ellipticus*	Elaeocarpaceae	Dominant tree	Birds	59	64	cpDNA	4688–4689	0.703	0.00031	W-E differentiation (Kinki)	Figure 1a, [3]
*Prunus zippeliana*	Rosaceae	Tree	Birds	73	83	cpDNA	3838–3883	0.661	0.00084	Unique type in Kyushu and Ryukyu	Figure 1b, [3]
*Myrsine seguinii*	Myrsinaceae	Tree	Birds	22	22	cpDNA	2625–2676	0.506	0.00212	W-E differentiation (Kinki to Shikoku)	Figure 1c, [27]
*Photinia glabra*	Rosaceae	Tree	Birds	42	129	cpDNA	4062–4087	0.693	0.00046	W-E differentiation (Chugoku)	Figure 1d, [35]
*Daphne kiusiana*	Thymelaeaceae	Shrub	Birds	19	19	cpDNA	3378–3387	0.414	0.00027	Unique type in Kyushu	[3]
*Alpinia japonica*	Zingiberaceae	Perennial herb	Birds	46	48	cpDNA	2919–2945	0.605	0.00110	No clear structure	[3]
*Arachniodes sporadosora*	Dryopteridaceae	Fern	Wind	33	35	cpDNA	349–357	-	-	No clear structure	[3]
*Arachniodes aristata*	Dryopteridaceae	Fern	Wind	41	41	cpDNA	628–662	-	-	No clear structure	[3]
*Castanopsis sieboldii*	Fagaceae	Dominant tree	Dropping, animals, birds	40	937	EST-SSR	-	0.631	-	W-E differentiation, unique cluster in Ryukyu	Figure 1e, [37]
*Castanopsis cuspidata*	Fagaceae	Dominant tree	Dropping, animals, birds	17	368	EST-SSR	-	0.741	-	No clear structure	[37]
**Insects**											
*Curculio hilgendorfi*	Curculionidae	Weevil	Flying but low dispersability	62	204	mtDNA	2709	0.969	0.00624	W-E differentiation (Kinki to Chugoku- Shikoku), unique clade in Ryukyu	Figure 1f, [32,33]
*Curculio sikkimensis*	Curculionidae	Weevil	Flying but low dispersability	33	115	mtDNA	971	0.933	0.00465	W-E differentiation (Kinki to Chugoku- Shikoku)	Figure 1g, [32,34]
*Rhynchaenus dorsoplanatus*	Curculionidae	Weevil	Flying but low dispersability	55	171	mtDNA	2343	0.973	0.0015	Unique clade in Kyushu	Figure 1h, [36]

**Table 2:** The observed genetic gap between the western and eastern parts of Japan revealed by phylogeographic studies from terrestrial plant and animal species [54,57–79].

**Class**	**Species**	**Family**	**Common name**	**Vegetation zone**	**Geographic gap**	**References**
Plants	*Pinus thunbergii*	Pinaceae	Black pine	Warm temperate zone	Kinki to Chubu	[63]
	*Zanthoxylum ailanthoides*	Rutaceae	Prickly ash	Warm temperate zone	Chugoku-Shikoku	[78]
	*Abies firma*	Pinaceae	Fir	Warm and cool temperate zone	Kinki to Chubu	[73]
	*Cerasus jamasakura*	Rosaceae	Cherry	Warm and cool temperate zone	Kyushu-Chugoku	[71]
	*Chamaecyparis obtusa*	Cupressaceae	Cypress	Warm and cool temperate zone	Kinki to Chubu	[72]
	*Fagus crenata*	Fagaceae	Beech	Cool temperate zone	Kinki to Chugoku- Shikoku	[57]
	*Fagus japonica*	Fagaceae	Beech	Cool temperate zone	Kinki to Chubu	[58]
	*Quercus mongolica* var. *crispula*	Fagaceae	Oak	Cool temperate zone	Chubu	[60,66]
	*Quercus serrata*	Fagaceae	Oak	Cool temperate zone	Chubu	[66]
	*Carpinus japonica*	Betulaceae	Hornbeam	Cool temperate zone	Kinki to Chugoku- Shikoku	[59]
	*Carpinus tschonoskii*	Betulaceae	Hornbeam	Cool temperate zone	Chugoku-Shikoku	[59]
	*Carpinus laxiflora*	Betulaceae	Hornbeam	Cool temperate zone	Kinki-Chugoku	[79]
	*Magnolia hypoleuca*	Magnoliaceae	-	Cool temperate zone	Kinki-Chugoku	[79]
	*Aesculus turbinata*	Hippocastanaceae	Horse chestnut	Cool temperate zone	Kinki	[69]
	*Corylopsis*	Hamamelidaceae	-	Cool temperate zone	Kinki	[75]
	*Viola eizanensis*	Violaceae	Violet	Cool temperate zone	Chugoku-Shikoku	[70]
	*Carex conica*	Cyperaceae	Sedge	Cool temperate zone	Kinki-Chugoku	[76]
	*Cardamine scutata*	Brassicaceae	Herb	Cool temperate and boreal zone	Chubu to Tohoku	[62]
Insects	*Xylosandrus crassiusculus*	Scolytidae	Ambrosia beetle	Cool temperate zone	Chubu	[54]
	*Plateumaris sericea*	Chrysomelidae	Leaf beetle	Cool temperate zone	Kinki	[68]
Mammals	*Cervus nippon*	Cervidae	Sika deer	Warm and cool temperate zone	Chugoku	[64]
	*Sus scrofa*	Suidae	Wild boar	Warm and cool temperate zone	Chugoku to Chubu	[74]
	*Macaca fuscata*	Cercopithecidae	Macaque	Warm and cool temperate zone	Kinki to Chugoku- Shikoku	[61]
	*Lepus brachyurus*	Leporidae	Hare	Cool temperate zone	Kinki to Chugoku- Shikoku	[65]
	*Petaurista leucogenys*	Sciuridae	Giant flying squirrel	Cool temperate zone	Kinki to Chugoku- Shikoku	[67]
	*Ursus thibetanus*	Ursidae	Black bear	Cool temperate zone	Kinki	[77]
